# The urgent need for a multi-disciplinary core outcome set for the reporting of obstetric antiphospholipid antibody syndrome

**DOI:** 10.1186/1745-6215-16-S3-P4

**Published:** 2015-11-24

**Authors:** Srividhya Sankaran, May Ching-Soh, Catherine Nelson-Piercy

**Affiliations:** 1 Guy’s and St. Thomas’ NHS Foundation Trust, London, UK; 2 Women’s Centre, John Radcliffe Hospital, Oxford University Hospitals NHS Trust, Oxford, UK; 3 Queen Charlotte’s & Chelsea Hospital, Imperial College Healthcare NHS Trust, London, UK; 4 Women’s Health Academic Group, King’s Health Partners, London, UK

## Background

Obstetric antiphospholipid antibody syndrome (OAPS) is associated with adverse pregnancy outcomes. A systematic review (2004) demonstrated diversity in published studies, making it difficult to interpret the outcomes in various subgroups with OAPS.

Clinicians (and patients) are divided about the efficacy of any treatment, given the heterogeneity of the patient populations studied, endpoints, outcome measures reported and treatments used. Many of these patients are being cared for by their rheumatologist, haematologist, obstetrician and fertility specialists – who may have different recommendations concerning the need for low-molecular weight heparin (LMWH). Women who have sought fertility treatment are finding it difficult to discontinue their LMWH despite the lack of evidence for its efficacy. The lack of consensus on therapeutic measures between various clinical specialities and patients often leads to poor patient experience. Patient involvement in the decision making process in these studies have not been reported; it could be the prime motivation for clinicians to continue prescribing LMWH despite the lack of demonstrable efficacy. Hence we need to develop a core outcome set so that clinicians from various subspecialities and patients will be able to assess each of these studies in a standardised fashion.

## Methods

1. A systematic literature review to produce a comprehensive list of all maternal and neonatal outcomes.

2. A Delphi technique to modify the list of outcome measures into a core set agreed by various subspecialties and patients.

## Results

This proposed project is in the preparation stage. International collaboration with various subspecialties is planned. This project will benefit from the support and recommendations of COMET initiative experts.

## Conclusion

A consensus on core outcome set for reporting for OAPS, involving clinicians across various subspecialties (rheumatology, obstetrics, maternal-fetal medicine, fertility and haematologists) and the patients is essential for studying the effectiveness of various treatment modalities.

